# Phenotypic Changes in Growth-Arrested T Cell Hybrids: A Possible Avenue to Produce Functional T Cell Hybridoma

**DOI:** 10.3389/fimmu.2014.00229

**Published:** 2014-05-19

**Authors:** Koichi Kubota, Kazuya Iwabuchi

**Affiliations:** ^1^Department of Microbiology, Kitasato University School of Medicine, Sagamihara, Kanagawa, Japan; ^2^Department of Immunology, Kitasato University School of Medicine, Sagamihara, Kanagawa, Japan

**Keywords:** BW5147, cell growth-arrest, induction of differentiation, lymphocyte differentiation, reprograming, YACUT

Attempts to immortalize effector T cells by fusion of normal T cells with T cell lymphomas have been extensively made after the successful establishment of the B cell hybridoma that made it possible to infinitely produce monoclonal antibodies (1, 2). However, T cell hybridomas, which were mostly produced by using the T cell lymphoma line, BW5147, have contributed to T cell biology with limited success, because immortalizing effector T cells by cell fusion dose not necessarily result in immortalizing the *bona fide* effector functions of parental T cells. Meanwhile, T cells have been becoming recognized as consisting of more diverse populations than previously anticipated (3–6). Furthermore, a new class of lymphocytes termed innate lymphoid cells has been identified; they lack rearranged antigen receptors and are involved not only in immunity but also in inflammation and in tissue remodeling and repair (6–9). Thus, in order to gain a better understanding of the functions of various lymphocytes and their roles in tissue microenvironment, it is desirable to produce functional hybridomas that preserve the effector functions of the parental lymphocytes faithfully. We briefly review phenotypic changes in T cell hybrids from the point of view of somatic cell genetics and describe the concepts and principles behind devising an improved method for immortalizing various lymphocyte functions.

## Genetics of Somatic Cell Fusion

Somatic cell hybridization has been used over several decades to study the genetic basis of cellular differentiation (10, 11). Those studies revealed that intertypic cell hybrids of equal ploidity fail to express the tissue-specific differentiated traits of either parent, a phenomenon termed extinction (12), that activation of previously silent genes occurs in hybrids with biased gene dosages, and that re-expression of previously extinguished phenotypes can occur in the hybrid segregants that have eliminated chromosomes of one parental cell. The differentiated state of somatic cells is therefore thought to be regulated via trans-acting regulatory factors and maintained by continuous regulatory circuits (13).

It has recently been shown that fusion of pluripotent embryonic stem cells (ESCs) with differentiated somatic cells yields pluripotent hybrid cells, indicating that ESCs have the ability to reset or reprogram the epigenomes of somatic cell nuclei toward pluripotency, albeit at low frequencies, but not to completely identical levels with original ESCs (14–18). Accordingly, extinction and/or activation of tissue-specific genes in somatic cell hybrids may be elucidated by partial reprograming of somatic cell epigenomes (19). In fact, in heterokaryons formed by fusion between somatic cells and non-dividing myotubes, trans-acting factors have been shown to partially reprogram one parental cell epigenome toward another parental cell epigenome in a bi-directional manner depending on biased gene dosages (20–22). However, because previously extinguished phenotypes in growing hybrids can be re-expressed in hybrid segregants (10, 23), the epigenetic state of each differentiated cell genome is considered to be stably maintained in proliferating hybrids. In continuously proliferating hybrids, therefore, reprograming of one parental epigenome toward another parental epigenome appears to be less likely to occur than in non-dividing heterokaryons. The mechanisms of epigenetic inheritance during the cell cycle (24) and the cell cycle stage of hybrid parental cells (25, 26) may inevitably affect reprograming of somatic nuclei.

In contrast to intertypic cell hybrids, hybrids between the same cell types (intratypic hybrids) continue to express the tissue-specific products of both parental cells (10, 27). When intratypic fusions are conducted between cells whose maturation stages are different, resultant hybrids usually express a variety of phenotypes (10). This phenotypic diversity, as suggested by the epigenetic study on CD4^+^ T cell differentiation (28), may reflect intricate epigenetic changes that occur during the maturation processes in the same cell lineage. The T cell hybridomas that were hitherto produced by fusion of immature lymphomas, like BW5147, with differentiated T cells are considered to be such intratypic hybrids.

However, T cell hybridomas were in most cases isolated by selecting proliferating cells based on a particular trait from effector T cell phenotypes, e.g., antigenic responsiveness, production of a lymphokine or cytolytic activity, without thorough cytogenetic analyses. Thus, the properties expressed in some of the hybridomas so far reported might represent the traits of hybrids with unequal ploidity or the traits of the segregants that have eliminated or duplicated chromosomes of one parental cell.

## Properties of T Cell Hybridomas Produced by Cell Fusion between BW5147 Lymphoma and Normal T Lymphocytes

The BW5147/helper T cell hybridomas that expressed the T cell receptor (TCR) of both parental origins produced IL-2 upon engagement of the TCR with its cognate ligands (29, 30). On the other hand, BW5147 cells produced IL-2 by themselves when stimulated with calcium ionophore and phorbol ester mimicking the activation signals delivered when TCRs are engaged with cognate antigen (31). Furthermore, even cytolytic BW5147 hybridomas produced by fusion with cytotoxic T cells (CTLs) secreted IL-2 (32, 33) although CTLs usually had no ability to produce IL-2. Thus, the ability of hybridomas to produce IL-2 is most likely attributed to a property of the parental BW5147 lymphoma. Nevertheless, because of this ability, BW5147/T cell hybridomas – especially through the use of TCR-deficient BW5147 variants (34–36) – have made significant contributions to dissecting the structure and specificity of the TCR and also to analyzing antigen presentation by multiple types of antigen-presenting cells (37–41).

BW5147/T cell hybridomas have also contributed to the characterization of lymphokines, particularly the T cell replacing factor or eosinophil differentiation factor, now called IL-5 (42–45). However, because BW5147 lymphoma itself expresses a variety of lymphokines, including suppressor factors (31, 46, 47), it is necessary to be cautious when interpreting the results obtained with these lymphokine-producing hybridomas.

Fusion of BW5147 with CTLs usually yielded non-cytolytic hybridomas in which CD8α gene expression was suppressed by a trans-acting regulator derived from BW5147. Despite this, BW5147/T cell hybridomas with cytolytic activity were produced (32, 48), and their cytolytic activity was subsequently ascribed to the Fas/FasL cytolytic pathway (49). Because the Fas/FasL cytolytic activity may be weekly expressed by the BW5147 lymphoma itself (50), BW5147/T cell hybridomas might be permissive for the expression of the Fas/FasL cytolytic pathway, but not the perforin-mediated cytolytic pathway of parental CTLs (51).

Cytolytic BW5147/T cell hybrids, which grow in an IL-2-dependent manner were also produced (52), but when autonomously growing hybridomas were derived from them, they lost not only their dependence on IL-2, but also all other attributes of the CTL phenotypes (53) due to epigenetic mechanisms, not chromosomal segregation (54). In proliferating hybrids, all the gene expression programs of one parental cell genome that governs proliferation of the hybrid may conspire to determine the phenotype of the hybrid for unknown mechanisms. The above report is also reminiscent of the phenomenon termed “phenotypic exclusion” shown in melanoma/hepatoma hybrids (23).

In summary, BW5147/T cell hybridomas have considerably helped analyze certain aspects of T cell biology. However, since BW5147 genome simultaneously imposes, albeit not completely, its own programing on the hybridomas, BW5147 dose not faithfully immortalize effector functions of T cells.

## Production of Growth-Arrested Hybrids through Fusion of Lymphomas and Terminally Differentiated T Cells

The quiescent state of non-dividing differentiated cells is actively controlled by regulatory mechanisms (55, 56). When terminally differentiated cells are fused with tumor cells in the same cell lineage, the regulatory mechanisms may operate dominantly over tumor transformation mechanisms (57). This notion has been substantiated by a study showing that fusion of lymphomas such as EL4, S1A, and YACUT, with antigen-responsive T cell line G4 yielded hybrid cells that ceased to proliferate (58–60). These growth-arrested hybrids proliferate by antigenic stimulation and thus have a phenotype similar to that of the G4 parental cells, which divide several times by antigenic stimulation and then enter into the quiescent phase of the cell cycle.

Upon antigenic stimulation, T lymphocytes proliferate, differentiate, and mature into effector cells. This is followed by either cessation of proliferation to become memory cells or initiation of apoptotic cell death to avoid over-immune-responses, which are harmful for the body. Thus, we can infer from the above experiments that when lymphomas are fused with normal activated lymphocytes, resultant hybrids will cease to proliferate or undergo cell death, though not necessarily always. This scenario explains why attempts to produce hybridomas between normal T cells and a variety of lymphomas were in many cases unsuccessful (47).

## Unique Properties of the Hybrids between YACUT Lymphoma and Differentiated Lymphocytes

YACUT lymphoma derives from a Moloney leukemia virus (MoLV)-induced lymphoma originating from a T cell in an immature stage of development (61, 62). In growth-arrested YACUT/G4 hybrids, the G4 cell genome introduced into the YACUT lymphoma suppressed the immature phenotypes as well as the transformed phenotype of YACUT and imposed its own programing of terminally differentiated traits on the hybrids (62). Unlike growth-arrested EL4/G4 and S1A/G4 hybrids, prolonged growth of growth-arrested YACUT/G4 hybrids resulted in the appearance of continuously proliferating cells with the differentiated phenotype due to an increase in the number of the tumor-derived chromosome 15 carrying the MoLV-inserted *pvt-1* gene (62). Furthermore, no segregants expressing immature phenotypes appeared after prolonged propagation of the re-transformed hybrids. From these observations, we can hypothesize that the epigenome of immature lymphoma nuclei differentiates into the mature epigenetic state of the G4 cell nuclei in the growth-arrested hybrids. This concept of association of cell growth-arrest with the induced differentiation of the immature lymphoma in lymphoma/lymphocyte hybrids (62) is parallel to the notion that tumor/normal cell hybrids in which tumor malignancy is suppressed are executing the differentiation program of a normal parental cell under the microenvironment *in vivo* (63, 64). This hypothesis predicts that fusion of the YACUT lymphoma with T lymphocytes followed by re-transformation will generate functional T cell hybridomas.

On the basis of this prediction, we have produced several functional hybridomas as summarized in Table 1. From rapidly dying growth-arrested hybrids produced by fusion of YACUT with a secondary mixed-lymphocyte culture cell, cytolytic hybridomas expressing the natural killer (NK) cell activation and inhibitory receptors were produced (65–67). From long-lived growth-arrested hybrids of the same fusion, we obtained another hybridoma termed B6HO3, which formed cell conjugates with bacteria-infected macrophages and produced IFN-γ in response to IL-18 secreted from dying macrophages caused by bacterial infection (68, 69). The parental αβ T cell of this hybridoma was a cell that belongs to minor innate-like αβ T cells. This B6HO3 hybridoma has helped reveal that subsets of innate lymphocytes respond to macrophage cell death caused by bacterial infection with the production of innate IFN-γ, which plays an important defensive role at an early stage of bacterial infections (70, 71).

**Table 1:** **Summaries of functional hybridomas produced from growth-arrested YACUT/lymphocyte hybrids**.

Fusion	Growth-arrest after fusion	Growth stimulatory antigen or cytokine	Transformed cell (designation)	Characteristics of hybridoma (reference)	Parental cell
YACUT × G4	Yes	Splenocytes with Mls^a^, H-2^b^, or H-2^d^ antigen	G6g1, E4e1	T cell helper activity (60, 62)	Mls^a^-responsive T cell line G4
YACUT × 2^0^MLC	Yes (rapid cell death without stimulation)	DBA/2 splenocytes	3A2, 7D4	Non-MHC-restricted cytotoxicity (65). Activation and inhibitory receptors^+^ (66, 67)	CTL
YACUT × 2^0^MLC	Yes	DBA/2 splenocytes	B6HO3	IFN-γ production in response to IL-18 produced by dying bacteria-infected macrophages (68, 69)	Innate-like αβ T cell
YACUT × 2^0^MLC	Yes	Irradiated splenocytes	CB1	Requirement of TNF-α for the growth in the presence of irradiated splenocytes CD3^−^, TCR^−^	Innate lymphoid cell?
YACUT × iNKT	Yes	IL-7 + IL-15	F3a	Growth in longitudinal cell clusters; CD244.1 and 2^+^	iNKT cell

*2^0^MLC: secondary C3H anti-DBA/2 mixed-lymphocyte culture*.

Another hybridoma termed CB1 was also produced; its membrane phenotype was CD3^−^, TCR^−^, B220^−^, CD19^−^, CD244^+^, and FcRII/III^+^, suggesting that the parental cell was a NK-like cell or a cell belonging to innate lymphoid cells; CB1 was of interest, because the proliferation of the cells was suppressed in the presence of irradiated spleen cells and TNF-α was intimately involved in this growth. Further, by fusing YACUT with invariant natural killer T (iNKT) cells and culturing the resultant growth-arrested hybrids in the presence of IL-7 and IL-15, we obtained hybridoma F3a, which was unique in that it proliferated in longitudinal cell clusters. The functions of CB1 and F3a and their parental lymphocyte functions remain to be determined by further studies, but insofar as our prediction is valid, previously unknown functions of lymphocytes will be unveiled through these hybridomas.

## Concluding Remarks

Fusion of lymphomas with normal lymphocytes yields mostly growth-arrested hybrids in which epigenomes of immature lymphomas are thought to be induced to differentiate toward the mature epigenetic state of normal lymphocyte nuclei. Thus, if we devise some methods of making the growth-arrested hybrids re-proliferate indefinitely, it will make it possible to immortalize effector functions of normal lymphocytes. In this respect, the growth-arrested YACUT/normal T cell hybrids are of importance. After prolonged growth by repeated stimulation, they resume indefinite proliferation presumably because of chromosomal instability of the YACUT genome, thereby becoming functional hybridomas that exhibit the phenotypes of their parental lymphocytes. This YACUT hybridization system and further research based on our hypothesis will shed light on hitherto unknown functions of lymphocytes and contribute to a better understanding of lymphocyte biology.

## Conflict of Interest Statement

The authors declare that the research was conducted in the absence of any commercial or financial relationships that could be construed as a potential conflict of interest.
